# The association between assisted reproductive technology and cardiac remodeling in fetuses and early infants: a prospective cohort study

**DOI:** 10.1186/s12916-022-02303-6

**Published:** 2022-04-01

**Authors:** Wenjing Bi, Yangjie Xiao, Xin Wang, Li Cui, Guang Song, Zeyu Yang, Ying Zhang, Weidong Ren

**Affiliations:** 1 Department of Ultrasound, Shengjing Hospital of China Medical University, 36# of Sanhao St. Heping District, Shenyang, 110004 China; 2 Department of Ultrasound, Union Hospital, Tongji Medical College, Huazhong University of Science and Technology, Wuhan, China

**Keywords:** Assisted reproductive technology, Cardiac remodeling, Fetal echocardiography, Speckle-tracking, Tissue Doppler imaging

## Abstract

**Background:**

Limited data exist regarding the potential impact of assisted reproductive technology (ART) on cardiac remodeling. In particular, whether different ART techniques are related to different cardiac alterations remains unclear. We aimed to evaluate cardiac changes in fetuses and infants arising from ART and fetal cardiac alterations in fetuses conceived by specific ART procedures.

**Methods:**

This prospective and observational cohort study recruited 111 fetuses conceived by ART and 106 spontaneously conceived controls between December 2017 and April 2019. Echocardiography was performed between 28^+0^ and 32^+6^ weeks-of-gestation and at 0–2 and 6 months after birth.

**Results:**

A total of 88 ART fetuses and 85 controls were included in the final analysis. Compared to controls, ART fetuses demonstrated a globular enlarged left ventricle (LV) (LV sphericity index of mid-section, 2.29 ± 0.34 vs. 2.45 ± 0.39, *P* = 0.006; LV area, 262.33 ± 45.96 mm^2^ vs*.* 244.25 ± 47.13 mm^2^, *P* = 0.002), a larger right ventricle (RV) (RV area, 236.10 ± 38.63 mm^2^ vs*.* 221.14 ± 42.60 mm^2^, *P* = 0.003) and reduced LV systolic deformation (LV global longitudinal strain (GLS), −19.56% ± 1.90% vs*.* −20.65% ± 1.88%, *P* = 0.013; LV GLS rate S, −3.32 ± 0.36 s^-1^ vs. −3.58 ± 0.39 s^-1^, *P* = 0.023). There were no significant differences between the ART and control groups at postnatal follow-ups. Furthermore, we found fetal cardiac morphometry and function were comparable between different ART procedures. Compared to controls, the fetuses derived from various ART procedures all exhibited impairments in the LV GLS and the LV GLS rate S.

**Conclusions:**

Our analysis demonstrated that subclinical cardiac remodeling and dysfunction were evident in ART fetuses, although these alterations did not persist in early infancy. In addition, various ART procedures may cause the same unfavorable changes in the fetal heart.

**Trial registration:**

This trial was registered at the Chinese Clinical Trial Registry (www.chictr.org.cn) (ChiCTR1900021672) on March 4, 2019, retrospectively registered.

**Supplementary Information:**

The online version contains supplementary material available at 10.1186/s12916-022-02303-6.

## Background

Assisted reproductive technologies (ARTs) have allowed millions of infertile couples to achieve pregnancy. Current estimates state that over 8 million babies have been born by ART, with a significant upward trend over recent years [1]. Given the significant global uptake of ART, there are universal concerns relating to the potential impact of ART on the health of future generations.

According to the theory of fetal programming of cardiovascular disease [2, 3], there is a possibility that ART may predispose apparently healthy offspring to permanent reprogramming of cardiovascular development [4, 5]. However, only a handful of studies have focused on the effects of ART on cardiac changes. A previous cohort study of ART offspring demonstrated cardiac remodeling and dysfunction in utero that persisted into postnatal life [6, 7]. In another study, cardiac dysfunction was observed in ART children but not changes in cardiac morphometry [8]. Another study demonstrated right ventricle (RV) enlargement and diastolic dysfunction in ART pre-adolescents under high-altitude conditions [9]. It is clear that only limited data exist regarding the impact of ART on cardiac remodeling and that the exact pattern of cardiac changes remains controversial.

The evident differences between published studies could be explained by potential confounders, at least in part. For instance, ART is associated with an increased prevalence of advanced maternal age, twin pregnancy, prematurity, and low birth weight [10]; these factors are all known to be associated with cardiac remodeling or dysfunction [11–14]. On the other hand, the heterogeneity between ARTs used in different research studies might play a role in the poor consistency in previous findings. For example, Valenzuela-Alcaraz et al. [6] and von Arx et al. [9] both recruited cases that were conceived by in vitro fertilization (IVF) or intracytoplasmic sperm injection (ICSI), while Zhou et al. [15] also included children that were conceived by oocyte donation and artificial insemination. Liu et al. [8] and Xu et al. [16] only studied children that were conceived by IVF. Furthermore, the proportions of subjects conceived by frozen embryo transfer (FET) have also proven to be variable in previous studies.

Accordingly, we designed a prospective cohort study that was controlled for these potential confounding factors and investigated cardiac alterations that were related to ART in fetuses and infants. We also investigated cardiac changes in fetuses conceived by specific ART processes. Because cardiac dysfunction may be subtle, we used speckle-tracking echocardiography (STE) to measure deformation of the myocardium in ART offspring.

## Methods

### Study

**Fig. 1:**
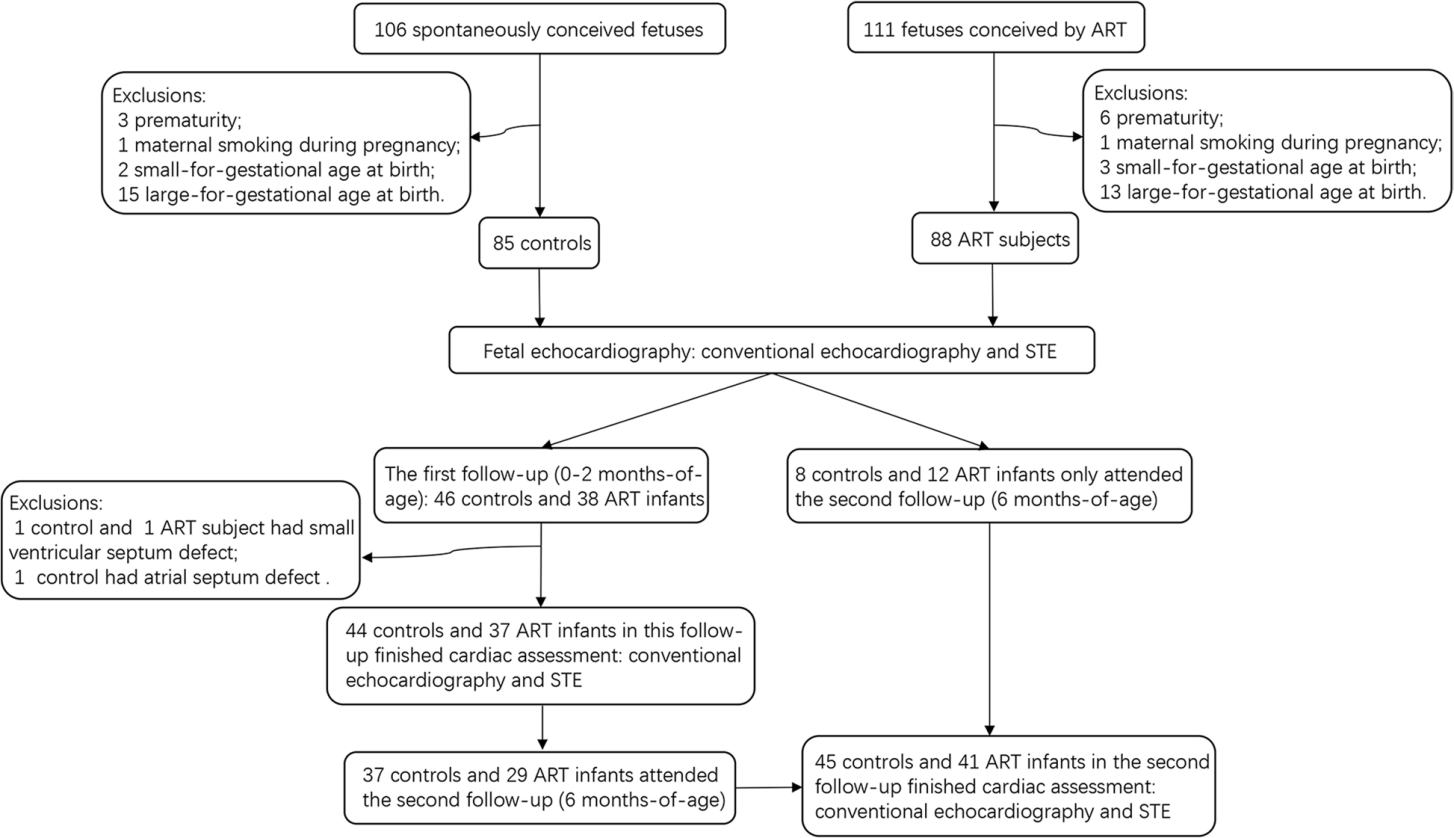
Participant flowchart. ART, assisted reproductive technology; STE, speckle-tracking echocardiography

This was a prospective and observational cohort study; the detailed protocol was published previously [17]. Initially, we recruited 111 fetuses that had been conceived by ART and 106 controls at the gestational age of 28^+0^ to 32^+6^ weeks between December 2017 and April 2019 at the Department of Ultrasound, Shengjing Hospital of China Medical University, Shenyang, China. All participants were singletons, and without any structural or chromosomal anomalies, evidence of infection, or any maternal medical diseases [17]. Twenty-three ART fetuses and 21 controls were excluded due to prematurity, smoking during pregnancy, or small/large-for-gestational age (Fig. 1). Data from 88 ART conceived fetuses and 85 controls were included in the final analysis.

The time points of the two follow-up visits were set at 0–2 and 6 months after birth. At each of the three visits, the participants underwent a standard protocol to receive cardiac assessment, including conventional echocardiography and STE. The Ethics Committee of Shengjing Hospital of China Medical University approved the study, and written informed consent was obtained from all parents. The trial registration number for the present study is ChiCTR1900021672.

### Fetal assessment

During prenatal evaluation, two-dimensional fetal ultrasound examinations were performed with an EPIQ 7C ultrasound system (Philips Ultrasound, Inc., Bothell, Washington) equipped with 3–5 MHz curved-array (C5-1) and 1–5 MHz sector-array (S5-1) probes. The same experienced operator (B-WJ) conducted all examinations, including the assessment of estimated fetal weight, placental Doppler, and fetal echocardiography [17].

Fetal echocardiography included a comprehensive examination to assess structural heart integrity, cardiac morphometry, and function, the latter was evaluated by both conventional echocardiography and STE analysis [17]. For STE, we acquired videos for offline analysis, including the apical or basal 4-chamber, long-axis, and 2-chamber views, as well as basal-, mid-, and apical-short-axis views. Images were analyzed by a single experienced operator (B-WJ) with QLAB software (10.8 version; Philips Healthcare, Andover, Massachusetts) [17]. The results of STE analysis included values for biventricular mid-myocardial global longitudinal strain (GLS), GLS rate, longitudinal time to peak (T2P) strain, and standard deviation of left ventricle (LV) longitudinal T2P strain, as well as LV mid-myocardial global circumferential strain (GCS), GCS rate, circumferential T2P strain, and standard deviation of circumferential T2P strain. We also generated bull’s-eye maps for LV longitudinal and circumferential strain. Global parameters were calculated by segmental averaging; for example, RV GLS was measured as the mean value of three lateral segments and the LV GLS and GCS of all 18 segments. To better compare T2P strain and LV dyssynchrony in different heart rates, all cardiac cycles were adjusted to 400ms.

### Postnatal assessment

Postnatal echocardiography, including conventional morphological and functional evaluations and STE analysis, was performed by the same skilled operator (B-WJ) using an EPIQ 7C ultrasound system (Philips Ultrasound, Inc., Bothell, Washington) equipped with a 5–12 MHz sector-array (12S) probe. Infants with congenital heart defects that were not diagnosed prenatally were excluded from the follow-up visits (Fig. 1).

### Statistical analysis

The IBM SPSS software (version 24; SPSS Inc., Chicago, IL) was used for analysis. Data are presented as mean ± standard deviation (SD), median (interquartile range), or percentage (number) where appropriate. Categorical variables were compared using the Pearson’s *χ*^2^ test, the continuity-adjusted *χ*^2^ test, or Fisher’s exact test. Comparisons of continuous variables between the ART and control groups were performed by independent *t* tests and adjusted for association to confounding factors by linear regression. Analysis of variance (ANOVA) was used to compare three groups with Bonferroni correction if *P* < 0.05. The Kruskal-Wallis test was used when we observed heterogeneity of variance between groups. Body surface area was calculated according to the Meban formula [18]. All *P* values were two-tailed, and *P* < 0.05 was considered statistically significant.

Inter-observer and intra-observer variability were assessed as previously described [17]. Speckle-tracking assessments were repeated in 15 prenatal and 15 postnatal randomly selected cases by a second observer (X-YJ) blinded to the group assignment and the prior measurement and by the first observer (B-WJ) after an interval of at least 3 months, respectively. Intraclass correlation coefficients (ICCs) were calculated for repeatability and reproducibility. ICCs > 0.80 were recognized as excellent and 0.60 to 0.80 as good.

## Results

### Cardiac changes in ART fetuses and infants

Baseline and perinatal characteristics were similar when compared between the ART and control groups, except for the higher prevalence of nulliparity (*P* < 0.001), lower maternal (*P* = 0.008) and paternal (*P* = 0.014) educational level, and higher rates of cesarean section (*P* = 0.001) in the ART group (Table 1). Maternal age, gestational age at delivery, and birth weight were all comparable between the two groups; these are all factors that are known to influence cardiac changes [11, 13, 14].

**Table 1:** Baseline and perinatal characteristics of the study population

Characteristics	SC (*n* = 85)	ART (*n* = 88)	*P*
**Maternal characteristics**			
Age (years)	33.0 (31.5, 36.0)	34.0 (31.0, 36.0)	0.793
BMI (kg/m^2^)	26.11±3.44	25.74 (24.06, 28.29)	0.500
Han, % (*n*)	85.88 (73)	86.36 (76)	0.927
Nulliparity, % (*n*)	56.47 (48)	89.77 (79)	**<0.001**
Family cardiovascular history, % (*n*)^a^	57.65 (49)	48.86 (43)	0.247
Low socioeconomic level, % (*n*)	18.82 (16)	28.41 (25)	0.138
University education, % (*n*)	87.06 (74)	70.45 (62)	**0.008**
**Paternal characteristics**			
Age (years)	34.8±4.3	35.7±4.1	0.165
BMI (kg/m^2^)	25.77±3.34	25.32±3.48	0.386
Han, % (*n*)	88.24 (75)	88.64 (78)	0.934
Cigarette smoker, % (*n*)	34.12 (29)	43.18 (38)	0.221
Family cardiovascular history, % (*n*)^a^	57.65 (49)	53.41 (47)	0.575
Low socioeconomic level, % (*n*)	5.88 (5)	13.64 (12)	0.087
University education, % (*n*)	85.88 (73)	70.45 (62)	**0.014**
**Fertility and ART characteristics**			
Infertility cause, % (*n*)			
Unexplained	-	12.50 (11)	-
Female	-	45.45 (40)	-
Male	-	18.18 (16)	-
Female + male	-	23.86 (21)	-
ART technique, % (*n*)			
IVF	-	54.55 (48)	-
ICSI	-	43.18 (38)	-
IVF+ICSI	-	2.27 (2)	-
Transferred embryos, % (*n*)			
1	-	25.00 (22)	-
2	-	73.86 (65)	-
3	-	1.14 (1)	-
FET, % (*n*)	-	76.14 (67)	-
**Pregnancy complications, % (*n*)**			
Preeclampsia	4.71 (4)	9.09 (8)	0.256
Gestational diabetes	23.53 (20)	18.18 (16)	0.386
Placenta previa	2.35 (2)	4.55 (4)	0.710
Obstetric cholestasis	0 (0)	0 (0)	1.000
Prenatal corticoid exposure	1.18 (1)	2.27 (2)	1.000
**Delivery data**			
Gestational age at delivery (week)	39.6±1.0	39.5±0.9	0.694
Cesarean section, % (*n*)	48.24 (41)	72.73 (64)	**0.001**
Male, % (*n*)	47.06 (40)	52.27 (46)	0.493
Birth length (cm)	51.0 (49.5, 52.0)	51.0 (50.0, 52.0)	0.559
Birth weight (g)	3380±280	3448±316	0.138
**Neonatal outcome, % (*n*)**			
Admission to NICU	1.18 (1)	0 (0)	0.491
Major neonatal morbidity^b^	0 (0)	0 (0)	1.000
Perinatal mortality	0 (0)	0 (0)	1.000

*ART* Pregnancy conceived by assisted reproductive technologies, *BMI* Body mass index, *FET* Frozen embryo transfer, *ICSI* Intracytoplasmic sperm injection, *IVF* In vitro fertilization, *NICU* Neonatal intensive care unit, *SC* Spontaneous conception

^a^Family cardiovascular history is defined as the existence of congenital heart disease, coronary disease, hypertension, diabetes, hypercholesterolemia, or stroke in men<55 years and women<65 years

^b^Major neonatal morbidity is defined as the existence of bronchopulmonary dysplasia, necrotising enterocolitis, intraventricular haemorrhage, periventricular leukomalacia, retinopathy, persistent ductus arteriosus, or sepsis in the first 28 days of life

**Fig. 2:**
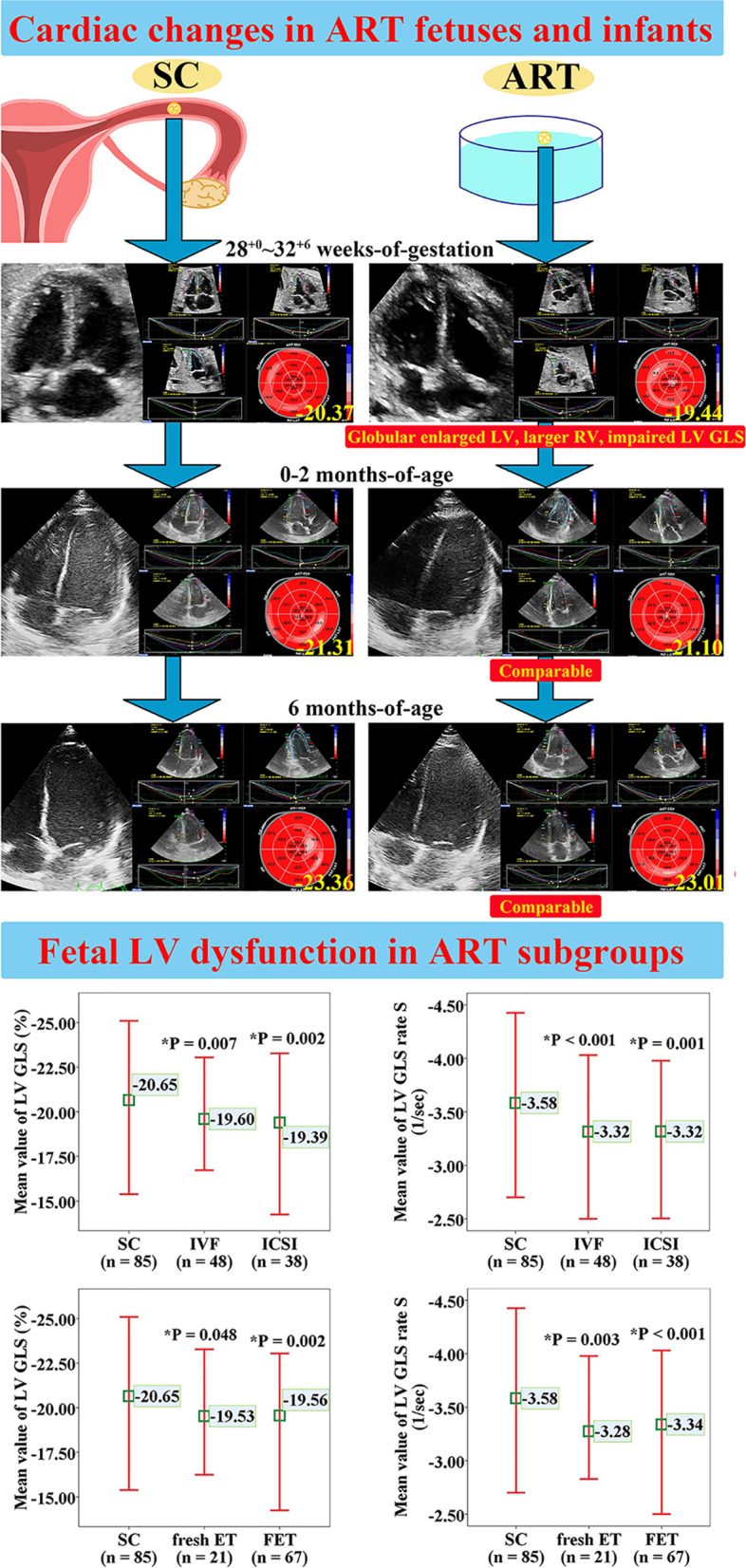
Study design and main findings. *Compared with the SC group by Bonferroni correction. ART, assisted reproductive technology; ET, embryo transfer; FET, frozen embryo transfer; GLS, global longitudinal strain; ICSI, intracytoplasmic sperm injection; IVF, in vitro fertilization; LV, left ventricle; RV, right ventricle; SC, spontaneous conception

Fetal assessment results are shown in Table 2. There was no significant difference in gestational age at ultrasound examination or estimated fetal weight when compared between groups. Placental Doppler parameters were similar between groups except for a lower middle cerebral artery pulsatility index (PI) (*P* = 0.013) and mean uterine artery PI (*P* = 0.010) in the ART group. Fetuses conceived by ART were associated with larger LV (*P* = 0.002) and RV (*P* = 0.003) areas and a lower LV sphericity index of mid-section (*P* = 0.006) (Fig. 2). Assessments of conventional echocardiographic function were similar when compared between the two groups, except for increased mitral E′ (*P* = 0.046) in ART fetuses. Deformation analysis for LV revealed that fetuses conceived by ART showed significant reductions in GLS (*P* = 0.013) (Fig. 2) and GLS rate S (*P* = 0.023) after adjusting for gestational age at scan, estimated fetal weight, parity, sex of offspring, gestational diabetes, mean uterine artery PI, frame rate, and LV area. With regard to RV STE analysis, there was no significant difference between the groups with respect to systolic and diastolic deformation.

**Table 2:** Fetal assessment of the study population

Characteristics	SC (*n* = 85)	ART (*n* = 88)	*P*	Adjusted P*
Gestational age at scan (week)	29.7 (28.7, 31.3)	29.7 (29.0, 30.8)	0.904	-
Estimated fetal weight (g)	1460 (1265, 1733)	1462 (1293, 1689)	0.683	-
Placental data				
Middle cerebral artery PI	1.98±0.31	1.90±0.32	0.080	**0.013**
Umbilical artery PI	1.04±0.17	1.03±0.16	0.565	0.736
Cerebroplacental ratio	1.89 (1.71, 2.12)	1.84 (1.62, 2.05)	0.332	0.080
Aortic isthmus PI	2.68±0.35	2.68±0.35	0.940	0.925
Ductus venosus PI	0.54 (0.45, 0.69)	0.55 (0.47, 0.73)	0.591	0.629
Mean uterine artery PI	0.85±0.21	0.74±0.20	**0.001**	**0.010**
**Cardiac morphometry**				
Left atrial area (mm^2^)	167.99±35.70	166.49±30.26	0.767	0.819
Right atrial area (mm^2^)	173.73±31.48	175.14±32.53	0.772	0.608
LV area (mm^2^)	244.25±47.13	262.33±45.96	**0.011**	**0.002**
RV area (mm^2^)	221.14±42.60	236.10±38.63	**0.016**	**0.003**
LVSI mid	2.45±0.39	2.29±0.34	**0.003**	**0.006**
LVSI base	2.50±0.40	2.41±0.34	0.081	0.276
RVSI mid	2.12 (1.97, 2.41)	2.15±0.32	0.229	0.326
RVSI base	2.11±0.28	2.16±0.26	0.229	0.187
Relative LV wall thickness	0.36±0.05	0.35±0.04	**0.035**	0.074
Relative interventricular septal thickness	0.37±0.06	0.36±0.05	0.134	0.347
Relative RV wall thickness	0.34±0.05	0.34±0.05	0.715	0.833
Cardiothoracic ratio	0.29 (0.27, 0.32)	0.29±0.03	**0.044**	0.051
**Systolic function**				
LVEF (%)	63.43±5.22	63.46±4.43	0.963	0.435
RVEF (%)	58.61±6.22	60.00 (55.95, 62.58)	0.995	0.915
Left stroke volume (ml)	2.46±0.71	2.25 (1.78, 3.06)	0.923	0.991
Right stroke volume (ml)	3.58 (3.01, 3.94)	3.69±0.95	0.702	0.564
Left cardiac output (ml/min)	352.25±97.65	328.69 (253.64, 419.31)	0.639	0.727
Right cardiac output (ml/min)	528.57 (446.15, 574.26)	537.22±131.70	0.738	0.548
Mitral ring displacement (mm)	6.75±1.81	5.69 (4.49, 7.79)	**0.025**	0.111
Tricuspid ring displacement (mm)	8.30±1.81	7.17 (6.25, 9.16)	**0.012**	0.105
Mitral S′ (cm/s)	5.74±0.82	5.80 (5.13, 6.29)	0.645	0.760
Tricuspid S′ (cm/s)	6.29 (5.71, 6.96)	6.09 (5.71, 6.77)	0.515	0.947
**Diastolic function**				
Mitral E/A	0.72±0.08	0.75±0.10	0.050	0.164
Tricuspid E/A	0.73 (0.68, 0.79)	0.73 (0.67, 0.80)	0.891	0.668
Mitral E deceleration time (ms)	40.00 (32.00, 48.00)	44.00 (32.00, 54.00)	0.548	0.362
Tricuspid E deceleration time (ms)	28.00 (21.00, 36.00)	29.00 (22.00, 39.50)	0.784	0.806
Mitral E′ (cm/s)	5.59±0.79	5.84±0.86	**0.047**	**0.046**
Mitral A′ (cm/s)	8.35±1.69	8.19±1.64	0.521	0.334
Tricuspid E′ (cm/s)	6.77 (5.61, 8.51)	6.68 (5.90, 8.49)	0.824	0.913
Tricuspid A′ (cm/s)	11.04±1.81	10.88±2.42	0.627	0.652
Left isovolumic relaxation time (ms)	48.16±9.44	45.89±8.57	0.098	0.138
**Global cardiac function**				
Left myocardial performance index	0.45±0.14	0.39 (0.31, 0.53)	0.725	0.478
Right myocardial performance index	0.38±0.12	0.38±0.16	0.988	0.707
**Deformation analysis**				
Frame rate (fps)	97.0 (92.0, 103.5)	95.0 (89.3, 103.0)	0.113	-
Heart rate (bpm)	145.1±7.1	144.2±6.5	0.379	0.181
Systolic deformation				
LV GLS (%)	−20.65±1.88	−19.56±1.90	**<0.001**	**0.013**
LV GLS rate S (s^-1^)	−3.58±0.39	−3.32±0.36	**<0.001**	**0.023**
LV global longitudinal T2P strain (ms)	209.60±13.92	214.50±14.40	**0.024**	0.119
LV GCS (%)	−19.83±2.49	−18.56 (−20.35, −17.20)	**0.031**	0.260
LV GCS rate S (s^-1^)	−3.89±0.56	−3.70±0.54	**0.026**	0.195
LV global circumferential T2P strain (ms)	208.72±12.95	206.91±12.90	0.359	0.794
RV GLS (%)	−21.58±3.64	−20.58±3.83	0.083	0.812
RV GLS rate S (s^-1^)	−3.41±0.76	−3.25±0.82	0.210	0.906
RV global longitudinal T2P strain (ms)	196.00 (185.00, 217.50)	202.33±25.47	0.983	0.410
Diastolic deformation				
LV GLS rate E (s^-1^)	3.22±0.47	3.16±0.44	0.383	0.623
LV GLS rate A (s^-1^)	3.85±0.57	3.63±0.52	**0.010**	0.100
LV GCS rate E (s^-1^)	3.53±0.51	3.37 (2.93, 3.72)	0.209	0.993
LV GCS rate A (s^-1^)	3.46±0.67	3.17±0.59	**0.004**	0.165
RV GLS rate E (s^-1^)	3.19±1.19	2.78 (2.08, 3.45)	0.203	0.235
RV GLS rate A (s^-1^)	3.60 (2.93, 4.30)	3.78±1.32	0.899	0.936
Dyssynchrony				
Longitudinal strain SD_t18S_ (ms)	26.41±6.59	27.28±6.45	0.380	0.321
Circumferential strain SD_t18S_ (ms)	22.75 (17.62, 27.74)	24.13±6.97	0.595	0.493

*ART* Pregnancy conceived by assisted reproductive technologies, *GCS* Global circumferential strain, *GLS* Global longitudinal strain, *LV* Left ventricle, *LVEF* Left ventricular ejection fraction, *LVSI* Left ventricular sphericity index, *PI* Pulsatility index, *RV* Right ventricle, *RVEF* Right ventricular ejection fraction, *RVSI* Right ventricular sphericity index, *SC* Spontaneous conception, *SD*_*t18S*_ Standard deviation of the time to peak systolic strain of 18 segments, *T2P* Time to peak

*P values calculated by linear regression adjusted for gestational age at scan, estimated fetal weight, parity, sex of offspring, gestational diabetes, and mean uterine artery PI. For LV/RV deformation parameters, *P* values were additionally adjusted for frame rate and LV or RV area. For placental data, *P* values were adjusted for gestational age at scan, estimated fetal weight, parity, sex of offspring, and gestational diabetes

The characteristics of the infants at first follow-up are shown in Additional file 1: Table S1 while the results of the anthropometry and cardiac assessment are shown in Additional file 1: Table S2. There was no significant difference in age or body surface area between groups at the time of the scan. Results derived from conventional echocardiography were comparable between the ART and control groups, with except for a thinner relative RV wall thickness (*P* = 0.028) and higher tricuspid E/A (*P* = 0.046) in infants born by ART. In addition, there were no significant differences in deformation analysis when compared between the two groups.

The characteristics of the infants at second follow-up are shown in Additional file 1: Table S3. The results derived from anthropometry and cardiac assessment are presented in Additional file 1: Table S4. The age at evaluation was similar between the two groups, whereas body surface area was higher (*P* < 0.001) in infants born via ART. There was no significant difference between the two groups with respect to conventional echocardiographic parameters and deformation analysis (Fig. 2).

### Cardiac alterations of fetuses conceived by specific ART techniques

The baseline characteristics of fetuses conceived by specific ART procedures are summarized in Additional file 1: Tables S5 and S6. There was no significant difference in cardiac morphometry and function when compared between IVF and ICSI groups or between fresh embryo transfer (ET) and FET groups (Fig. 2 and Additional file 1: Tables S7 and S8). Compared with controls, IVF fetuses showed a lower mean uterine artery PI (*P* = 0.031), larger RV (*P* = 0.038), lower LV sphericity index of mid-section (*P* = 0.016) and of base-section (*P* = 0.035) and a decreased LV GLS (*P* = 0.007), LV GLS rate S (*P* < 0.001), and LV GCS (*P* = 0.039). ICSI cases demonstrated a lower mean uterine artery PI (*P* = 0.019), decreased mitral (*P* = 0.021) and tricuspid ring displacement (*P* = 0.027), higher mitral E’ (*P* = 0.036), along with decreased LV GLS (*P* = 0.002), LV GLS rate S (*P* = 0.001), and LV GCS rate A (*P* = 0.007) when compared to controls. Moreover, the fresh ET group was associated with a lower LV GLS (*P* = 0.048), LV GLS rate S (*P* = 0.003), and LV GCS rate S (*P* = 0.044), when compared to fetuses conceived spontaneously. In the FET group, fetuses showed a lower mean uterine artery PI (*P* = 0.001) and LV sphericity index of mid-section (*P* = 0.005), larger LV (*P* = 0.012) and RV (*P* = 0.035), decreased relative LV wall thickness (*P* = 0.031) and mitral (*P* = 0.017) and tricuspid ring displacement (*P* = 0.011), impaired LV GLS (*P* = 0.002), LV GLS rate S (*P* < 0.001), LV GLS rate A (*P* = 0.046), and LV GCS rate A (*P* = 0.022), as compared to controls. Collectively, these data show that the common features of cardiac change in ART subgroups were a reduction in LV GLS and LV GLS rate S (Fig. 2).

### Intra- and inter-observer variability

With regard to the intra- and inter-observer variability of prenatal measurements, we found that the ICCs were excellent or good for LV deformation parameters. With regard to the intra- and inter-observer variability of postnatal measurements, we found that the ICCs were excellent for almost all LV deformation parameters (Additional file 1: Table S9).

## Discussion

In this prospective and observational cohort study, we found that subclinical changes associated with cardiac morphology and function were detectable in ART fetuses. However, these alterations did not persist in the early infantile period (Fig. 2).

Consistent with previous studies showing cardiac morphological changes in ART fetuses [6, 19], in the present study, we demonstrated a globular enlarged LV and a larger RV in ART fetuses. From a pathophysiological perspective, a spherical dilated shape will weaken the contractile properties of the ventricle [20, 21]. Indeed, the results of STE analysis showed that there was reduced left systolic function in ART fetuses. This systolic dysfunction was presented as significant reductions in LV GLS and GLS rate S. These findings partially agreed with those of Valenzuela-Alcaraz et al. [6] who reported impaired LV and RV systolic function in fetuses conceived by ART. However, consistent with our present findings, von Arx et al. [9] revealed similar RV systolic function in ART children and controls. With regard to diastolic performance, we observed increased mitral E’ in ART fetuses. However, previous studies described unchanged or reduced mitral E’ [6, 19]. Thus, the changes in mitral E’ that are reported here need to be further validated. Taken together, these data demonstrated that ART was associated with a globular enlarged LV, a larger RV, and LV dysfunction during the prenatal period.

A previous study failed to detect cardiac remodeling in ART children at low attitude [9]. Moreover, at high attitude, Scherrer et al. [4] reported a comparable cardiac index and E/E’ ratio when compared between ART and control children. Our current data support these previous research studies. In our evaluations carried out in the first 0–2 months of life, we only noted a thinner relative RV wall thickness and a higher tricuspid E/A ratio. In view of the large number of evaluations carried out and the deformation parameters were primary outcomes, these limited findings may not be clinically important. Moreover, the cardiac data were comparable between ART infants and controls when evaluated at 6 months of life. However, our findings are inconsistent with a previous study [6] in that reported the most compelling data by far by demonstrating signs of cardiac remodeling in utero and that these changes persisted postnatally in ART offspring. When considering possible explanations for this discrepancy, it is important to acknowledge that the enrollment criteria of the present study were more stringent. According to the Barker hypothesis [2, 3], when fetuses endure adverse events, the cardiac changes may be more likely to appear later in life under the mediation of certain conditions (e.g., obesity and metabolic syndrome). It seems possible that the ART-associated cardiac remodeling disappeared after birth in our “low cardiovascular risk” infants born via ART.

Thus far, few researchers have studied cardiac changes in association with specific ART techniques. A previous study reported larger atria and globular ventricles in fetuses conceived by fresh ET compared to those by FET [22]. Most recently, Boutet et al. [19] demonstrated larger atria, thicker myocardial walls, and more impaired systolic function in a fresh ET group when compared to an FET population. In the present study, we found that cardiac morphology and function were comparable between IVF and ICSI groups as well as between fresh ET and FET groups. Compared with controls, however, all these ART subgroups were associated with a reduced LV GLS and LV GLS rate S which represents LV systolic dysfunction. Strain parameters, especially LV GLS, are considered to be independent predictors of adverse cardiovascular outcome in subjects at risk [23–25]. Thus, it is important and necessary to evaluate LV GLS and its potential association with ART.

The underlying mechanisms by which ART results in fetal cardiac remodeling are poorly understood. Some investigators have suggested that, as with growth restricted fetuses, the cardiac remodeling in ART fetuses may be a response to increased RV afterload with placental abnormalities [26]. Many placental abnormalities, particularly the vascular lesions, are associated with impaired utero-placental perfusion which can be noted by abnormal uterine artery Doppler waveforms [27–29]. In previous studies, however, no difference in utero-placental perfusion was detected between ART pregnancies and spontaneous pregnancies [30, 31]. Several recent studies demonstrated better utero-placental perfusion in pregnancies conceived by FET than those conceived spontaneously or by fresh ET [32–34]. In our present study, ART pregnancies also demonstrated a significantly lower mean uterine artery PI than those conceived spontaneously. After subgroup analysis, the lower mean uterine artery PI was found to be related to FET but not fresh ET. Our findings suggest that ART fetuses, especially those conceived by FET, are associated with better utero-placental perfusion. Thus, the role of placental hemodynamic function during cardiac remodeling in ART fetuses needs to be validated further.

Recently, epigenetic mechanisms have been proposed to play an important role in the cardiovascular dysfunction of ART offspring [4, 5]. In a previous study, Rexhaj et al. [35] demonstrated that vascular dysfunction in ART mice was related to altered methylation at the promoter of the gene encoding eNOS in the aorta. It has also been proposed that oxidative stress during ART procedures may also cause long-lasting cardiovascular dysfunction in the offspring [36]. A previous study detected increased levels of intracellular reactive oxygen species in the mesenteric resistance arteries of ART mice [37]. In humans, some oxidative stress-associated proteins were found to be differentially expressed in umbilical arteries collected from ART offspring [16]. However, the exact role of epigenetic modifications and oxidative stress in ART-induced cardiac alterations have yet to be elucidated. Future studies need to investigate the mechanisms that underlie these potential effects.

Several limitations in this study should be taken into consideration. First, the range of gestational ages, and the ages at scanning on each visit may be too broad. To minimize the influences of individual differences on cardiac structure and function, linear regression analysis was adjusted for confounding factors, such as gestational age, age at scan, estimated fetal weight, and body surface area. Second, we acknowledge that the present study might be underpowered as there was a 50% loss during the follow-up period. Third, we excluded a range of confounding factors during recruitment, including twin pregnancies, pregnancies with structural/chromosomal anomalies, evidence of infection or any maternal medical disease (e.g., asthma, chronic hypertension, diabetes mellitus, heart disease, HIV or hepatitis infection, lupus and thyroid disease), prematurity, small/large-for-gestational age, and maternal smoking during pregnancy. The baseline variables (e.g., maternal age, gestational age at delivery, birth weight, preeclampsia, and gestational diabetes) were comparable in ART and control fetuses. However, it remains uncertain whether the observed cardiac changes were influenced by some unknown confounding factors (e.g., vanishing twin syndrome). Finally, as a single-center cohort study with limited universality, the results should be extrapolated to the overall ART population with an appropriate degree of caution.

## Conclusions

In summary, we found that fetuses conceived by ART experienced cardiac remodeling and LV dysfunction, although these cardiac alterations did not persist in early infancy. Furthermore, the fetal cardiac changes were comparable between ART subgroups. Decreased LV GLS and GLS rate S were both evident in fetuses conceived by various ART procedures. Future studies should include long-term follow-up of these cardiac changes in ART offspring, especially with regard to the lower LV GLS.

## Supplementary Information

**Additional file 1: Table S1.** Baseline and perinatal characteristics of the study population who attended the first follow-up (0-2 months-of-age). **Table S2.** Anthropometric data and cardiac assessment of the study population at the first follow-up (0-2 months-of-age). **Table S3.** Baseline and perinatal characteristics of the study population who attended the second follow-up (6 months-of-age). **Table S4.** Anthropometric data and cardiac assessment of the study population at the second follow-up (6 months-of-age). **Table S5.** Baseline and perinatal characteristics of controls and fetuses conceived by IVF and ICSI. **Table S6.** Baseline and perinatal characteristics of controls and fetuses conceived by fresh ET and FET. **Table S7.** Fetal assessment of controls and fetuses conceived by IVF and ICSI. **Table S8.** Fetal assessment of controls and fetuses conceived by fresh ET and FET. **Table S9.** Intra-observer and inter-observer ICCs for LV deformation parameters.

## Data Availability

The datasets used and/or analyzed during the current study are available from the corresponding author on reasonable request.

## Abbreviations

ART: Assisted reproductive technology
ET: Embryo transfer
FET: Frozen embryo transfer
GCS: Global circumferential strain
GLS: Global longitudinal strain
ICCs: Intraclass correlation coefficients
ICSI: Intracytoplasmic sperm injection
IVF: In vitro fertilization
LV: Left ventricle
PI: Pulsatility index
RV: Right ventricle
STE: Speckle-tracking echocardiography
T2P: Time to peak
