# Supplementary material for: The association between assisted reproductive technology and cardiac remodeling in fetuses and early infants: a prospective cohort study
Source: BMC Med. 2022 Apr 1;20:104. doi: 10.1186/s12916-022-02303-6 (PMC8973576; doi:10.1186/s12916-022-02303-6)
Supplement: Supplementary file 1 — Additional file 1: Table S1. Baseline and perinatal characteristics of the study population who attended the first follow-up (0-2 months-of-age). Table S2. Anthropometric data and cardiac assessment of the study population at the first follow-up (0-2 months-of-age). Table S3. Baseline and perinatal characteristics of the study population who attended the second follow-up (6 months-of-age). Table S4. Anthropometric data and cardiac assessment of the study population at the second follow-up (6 months-of-age). Table S5. Baseline and perinatal characteristics of controls and fetuses conceived by IVF and ICSI. Table S6. Baseline and perinatal characteristics of controls and fetuses conceived by fresh ET and FET. Table S7. Fetal assessment of controls and fetuses conceived by IVF and ICSI. Table S8. Fetal assessment of controls and fetuses conceived by fresh ET and FET. Table S9. Intra-observer and inter-observer ICCs for LV deformation parameters. [file 12916_2022_2303_MOESM1_ESM.docx]

**Additional File 1**

Table S1. Baseline and perinatal characteristics of the study population who attended the first follow-up (0-2 months-of-age).

Table S2. ﻿Anthropometric data and cardiac assessment of the study population at the first follow-up (0-2 months-of-age).

Table S3. Baseline and perinatal characteristics of the study population who attended the second follow-up (6 months-of-age).

Table S4. Anthropometric data and cardiac assessment of the study population at the second follow-up (6 months-of-age).

Table S5. Baseline and perinatal characteristics of controls and fetuses conceived by IVF and ICSI.

Table S6. Baseline and perinatal characteristics of controls and fetuses conceived by fresh ET and FET.

Table S7. Fetal assessment of controls and fetuses conceived by IVF and ICSI.

Table S8. Fetal assessment of controls and fetuses conceived by fresh ET and FET.

Table S9. Intra-observer and inter-observer ICCs for LV deformation parameters.

Table S1. Baseline and perinatal characteristics of the study population who attended the first follow-up (0-2 months-of-age)

| Characteristics | SC (n=44) | ART (n=37) | P |
| --- | --- | --- | --- |
| **Maternal characteristics** |  |  |  |
| Age (y) | 33.5±3.0 | 34.4±3.0 | 0.181 |
| BMI (kg/m^2^) | 26.83±3.52 | 25.28 (23.83, 27.78) | 0.375 |
| Han, % (n) | 86.36 (38) | 86.49 (32) | 0.987 |
| Nulliparity, % (n) | 59.09 (26) | 89.19 (33) | **0.002** |
| Family cardiovascular history, % (n)﻿† | 63.64 (28) | 54.05 (20) | 0.382 |
| Low socioeconomic level, % (n) | 20.45 (9) | 27.03 (10) | 0.487 |
| University education, % (n) | 86.36 (38) | 67.57 (25) | **0.043** |
| **Paternal characteristics** |  |  |  |
| Age (y) | 34.3±4.4 | 35.9±3.8 | 0.099 |
| BMI (kg/m^2^) | 25.67±3.26 | 25.32±3.28 | 0.634 |
| Han, % (n) | 88.64 (39) | 89.19 (33) | 1.000 |
| Cigarette smoker, % (n) | 25.00 (11) | 40.54 (15) | 0.136 |
| Family cardiovascular history, % (n)﻿† | 61.36 (27) | 54.05 (20) | 0.507 |
| Low socioeconomic level, % (n) | 6.82 (3) | 21.62 (8) | 0.053 |
| University education, % (n) | 86.36 (38) | 72.97 (27) | 0.132 |
| ﻿**Pregnancy complications, % (n)** |  |  |  |
| Preeclampsia | 9.09 (4) | 5.41 (2) | 0.838 |
| Gestational diabetes | 29.55 (13) | 8.11 (3) | **0.016** |
| ﻿Placenta previa | 2.27 (1) | 5.41 (2) | 0.878 |
| Obstetric cholestasis | 0 (0) | 0 (0) | 1.000 |
| Prenatal corticoid exposure | 0 (0) | 2.70 (1) | 0.457 |
| **Delivery data** |  |  |  |
| Gestational age at delivery (wk) | 39.6±0.9 | 39.6±1.0 | 0.811 |
| Cesarean section, % (n) | 52.27 (23) | 72.97 (27) | 0.056 |
| Male, % (n) | 50.00 (22) | 59.46 (22) | 0.395 |
| Birth length (cm) | 50.9±2.0 | 51.0 (50.0, 52.0) | 0.916 |
| Birth weight (g) | 3334±293 | 3446±318 | 0.104 |

ART, pregnancy conceived by assisted reproductive technologies; BMI, body mass index, SC, spontaneous conception.

†Family cardiovascular history is defined as the existence of congenital heart disease, coronary disease, hypertension, diabetes, hypercholesterolemia or stroke in men<55 years and women<65 years.

Table S2. ﻿Anthropometric data and cardiac assessment of the study population at the first follow-up (0-2 months-of-age)

| Characteristics | SC (n=44) | ART (n=37) | P | Adjusted P* |
| --- | --- | --- | --- | --- |
| **Age at scan** (m) | 1.1 (1.0, 1.3) | 0.9±0.5 | 0.090 | - |
| **﻿Anthropometric data** |  |  |  |  |
| ﻿Height (cm) | 55.6 (54.4, 56.8) | 55.3±2.7 | 0.485 | - |
| ﻿Weight (g) | 4503±659 | 4558±803 | 0.734 | - |
| BSA (cm^2^) | 2653.40±251.30 | 2663.12±297.93 | 0.874 | - |
| ﻿**Cardiac morphometry** |  |  |  |  |
| Left atrial area (mm^2^) | 263.45±63.45 | 265.36±69.41 | 0.898 | 0.207 |
| Right atrial area (mm^2^) | 242.12±46.03 | 237.62±61.40 | 0.708 | 0.978 |
| LV area (mm^2^) | 509.45±92.74 | 521.21±99.62 | 0.584 | 0.288 |
| RV area (mm^2^) | 376.64±79.37 | 365.05±83.95 | 0.526 | 0.479 |
| LVSI mid | 2.15 (2.03, 2.25) | 2.10 (2.00, 2.32) | 0.611 | 0.132 |
| LVSI base | 1.75±0.18 | 1.72 (1.63, 1.87) | 0.363 | 0.532 |
| RVSI mid | 2.92±0.40 | 2.73±0.42 | **0.040** | 0.162 |
| RVSI base | 1.82±0.19 | 1.83±0.17 | 0.922 | 0.434 |
| Relative LV wall thickness | 0.26±0.04 | 0.26 (0.24, 0.28) | 0.297 | 0.612 |
| Relative interventricular septal thickness | 0.29±0.05 | 0.28 (0.26, 0.32) | 0.627 | 0.997 |
| Relative RV wall thickness | 0.22±0.06 | 0.20±0.05 | 0.328 | **0.028** |
| **Systolic function** |  |  |  |  |
| LVEF (%) | 66.33±5.81 | 66.19±5.74 | 0.914 | 0.666 |
| RVEF (%) | 68.53±5.91 | 64.90 (61.10, 73.15) | 0.482 | 0.972 |
| Left stroke volume (ml) | 7.23±2.16 | 6.76 (5.73, 8.34) | 0.898 | 0.715 |
| Right stroke volume (ml) | 5.93 (5.06, 7.02) | 6.57±2.18 | 0.544 | 0.866 |
| Left cardiac output (ml/min) | 1063.17±343.29 | 1073.90±282.65 | 0.880 | 0.453 |
| Right cardiac output (ml/min) | 882.19 (742.83, 1009.00) | 961.00±334.77 | 0.620 | 0.951 |
| Mitral ring displacement (mm) | 7.43±1.17 | 7.88±1.22 | 0.097 | 0.350 |
| ﻿Tricuspid ring displacement (mm) | 12.23±2.01 | 11.78±2.67 | 0.392 | 0.944 |
| ﻿Mitral S′ (cm/s) | 5.62 (5.25, 6.18) | 5.97±0.81 | 0.579 | 0.516 |
| ﻿Tricuspid S′ (cm/s) | 8.28±1.47 | 7.97 (7.24, 8.51) | 0.494 | 0.977 |
| **Diastolic function** |  |  |  |  |
| Mitral E/A | 1.00 (1.00, 1.23) | 1.03 (1.00, 1.44) | 0.404 | 0.650 |
| Tricuspid E/A | 1.73±0.81 | 2.13±1.08 | 0.073 | **0.046** |
| Mitral E deceleration time (ms) | 86.11±28.39 | 85.76±21.85 | 0.950 | 0.854 |
| Tricuspid E deceleration time (ms) | 88.82±22.79 | 83.08±19.68 | 0.234 | 0.635 |
| ﻿Mitral E′ (cm/s) | 8.53 (7.58, 10.58) | 8.57 (7.37, 9.97) | 0.526 | 0.561 |
| Mitral A′ (cm/s) | 9.44±2.80 | 9.76±3.31 | 0.639 | 0.276 |
| Tricuspid E′ (cm/s) | 10.25 (7.78, 17.08) | 10.20 (8.14, 15.70) | 0.940 | 0.767 |
| ﻿Tricuspid A′ (cm/s) | 11.74±3.95 | 11.60 (8.14, 14.05) | 0.798 | 0.929 |
| ﻿Left isovolumic relaxation time (ms) | 49.41±9.27 | 45.00 (40.00, 50.00) | **0.030** | 0.110 |
| **Global cardiac function** |  |  |  |  |
| Left myocardial performance index | 0.27±0.09 | 0.30±0.09 | 0.117 | 0.767 |
| Right myocardial performance index | 0.17±0.07 | 0.12 (0.07, 0.31) | 0.535 | 0.204 |
| **Deformation analysis** |  |  |  |  |
| Frame rate (fps) | 113.0 (107.0, 120.5) | 114.1±10.0 | 0.888 | - |
| Heart rate (bpm) | 147.0±17.7 | 146.1±14.8 | 0.808 | 0.283 |
| Systolic deformation |  |  |  |  |
| LV GLS (%) | -21.30±1.60 | -20.84±2.24 | 0.300 | 0.435 |
| LV GLS rate S (﻿ s^-1^) | -3.35 (-3.47, -3.16) | -3.42±0.29 | 0.444 | 0.690 |
| LV global longitudinal T2P strain (ms) | 209.39±19.23 | 209.14±18.75 | 0.953 | 0.247 |
| LV GCS (%) | -23.50±2.50 | -23.82±2.52 | 0.568 | 0.459 |
| LV GCS rate S (﻿ s^-1^) | -4.15±0.59 | -4.15±0.54 | 0.997 | 0.999 |
| LV circumferential T2P strain (ms) | 197.30±22.86 | 199.46±20.97 | 0.661 | 0.133 |
| RV GLS (%) | -28.62±3.94 | -27.05±3.45 | 0.063 | 0.281 |
| RV GLS rate S (﻿ s^-1^) | -4.59 (-5.36, -4.22) | -4.47±1.37 | 0.154 | 0.363 |
| RV global longitudinal T2P strain (ms) | 209.00±28.74 | 216.65±20.83 | 0.170 | 0.236 |
| Diastolic deformation |  |  |  |  |
| LV GLS rate E (﻿ s^-1^) | 5.08±1.24 | 4.66±1.09 | 0.111 | 0.921 |
| LV GLS rate A (﻿ s^-1^) | 3.72±0.87 | 3.98±0.92 | 0.193 | 0.187 |
| LV GCS rate E (﻿ s^-1^) | 5.47±1.07 | 5.77±1.29 | 0.252 | 0.150 |
| LV GCS rate A (﻿ s^-1^) | 4.17±1.13 | 3.73 (3.04, 5.15) | 0.836 | 0.604 |
| RV GLS rate E (﻿ s^-1^) | 4.93 (4.08, 6.95) | 5.18±2.15 | 0.479 | 0.450 |
| RV GLS rate A (﻿ s^-1^) | 5.25±1.76 | 5.80±1.89 | 0.180 | 0.211 |
| Dyssynchrony |  |  |  |  |
| Longitudinal strain SD_t18S_ (ms) | 27.59±5.97 | 26.97±5.70 | 0.637 | 0.990 |
| Circumferential strain SD_t18S_ (ms) | 22.73±8.77 | 22.00 (17.00, 27.00) | 0.638 | 0.777 |

ART, pregnancy conceived by assisted reproductive technologies; BSA, body surface area; GCS, global circumferential strain; GLS, global longitudinal strain; LV, left ventricle; LVEF, left ventricular ejection fraction; LVSI, left ventricular sphericity index; RV, right ventricle; RVEF, right ventricular ejection fraction; RVSI, right ventricular sphericity index; SC, spontaneous conception; SD_t18S_, standard deviation of the time to peak systolic strain of 18 segments; T2P, time to peak.

*P values calculated by linear regression adjusted for age at scan, BSA, parity, sex of offspring, gestational diabetes, and mean uterine artery pulsatility index. For LV/RV deformation parameters, P values were additionally adjusted for frame rate and LV or RV area.

Table S3. Baseline and perinatal characteristics of the study population who attended the second follow-up (6 months-of-age)

| Characteristics | SC (n=45) | ART (n=41) | P |
| --- | --- | --- | --- |
| **Maternal characteristics** |  |  |  |
| Age (y) | 33.0±2.9 | 34.0 (31.0, 37.0) | 0.059 |
| BMI (kg/m^2^) | 26.30±3.49 | 26.46±3.82 | 0.843 |
| Han, % (n) | 91.11 (41) | 87.80 (36) | 0.883 |
| Nulliparity, % (n) | 68.89 (31) | 92.68 (38) | **0.006** |
| Family cardiovascular history, % (n)﻿† | 62.22 (28) | 46.34 (19) | 0.140 |
| Low socioeconomic level, % (n) | 15.56 (7) | 26.83 (11) | 0.199 |
| University education, % (n) | 86.67 (39) | 70.73 (29) | 0.070 |
| **Paternal characteristics** |  |  |  |
| Age (y) | 33.7±4.3 | 36.0±4.4 | **0.016** |
| BMI (kg/m^2^) | 25.90±3.28 | 24.60±3.20 | 0.067 |
| Han, % (n) | 88.89 (40) | 90.24 (37) | 1.000 |
| Cigarette smoker, % (n) | 35.56 (16) | 43.90 (18) | 0.429 |
| Family cardiovascular history, % (n)﻿† | 57.78 (26) | 51.22 (21) | 0.542 |
| Low socioeconomic level, % (n) | 4.44 (2) | 14.63 (6) | 0.210 |
| University education, % (n) | 86.67 (39) | 70.73 (29) | 0.070 |
| ﻿**Pregnancy complications, % (n)** |  |  |  |
| Preeclampsia | 6.67 (3) | 4.88 (2) | 1.000 |
| Gestational diabetes | 31.11 (14) | 12.20 (5) | **0.035** |
| ﻿Placenta previa | 4.44 (2) | 4.88 (2) | 1.000 |
| Obstetric cholestasis | 0 (0) | 0 (0) | 1.000 |
| Prenatal corticoid exposure | 0 (0) | 0 (0) | 1.000 |
| **Delivery data** |  |  |  |
| Gestational age at delivery (wk) | 39.6±0.8 | 39.5±0.8 | 0.634 |
| Cesarean section, % (n) | 53.33 (24) | 65.85 (27) | 0.238 |
| Male, % (n) | 44.44 (20) | 56.10 (23) | 0.280 |
| Birth length (cm) | 50.9±2.0 | 51.0 (50.0, 52.0) | 0.935 |
| Birth weight (g) | 3327±273 | 3483±318 | **0.017** |

ART, pregnancy conceived by assisted reproductive technologies; BMI, body mass index; SC, spontaneous conception.

†Family cardiovascular history is defined as the existence of congenital heart disease, coronary disease, hypertension, diabetes, hypercholesterolemia or stroke in men<55 years and women<65 years.

Table S4. Anthropometric data and cardiac assessment of the study population at the second follow-up (6 months-of-age)

| Characteristics | SC (n=45) | ART (n=41) | P | Adjusted P* |
| --- | --- | --- | --- | --- |
| **Age at scan** (m) | 6.3 (6.1, 6.5) | 6.4±0.3 | 0.456 | - |
| **﻿Anthropometric data** |  |  |  |  |
| ﻿Height (cm) | 68.6±5.2 | 70.6±2.1 | **0.020** | - |
| ﻿Weight (g) | 8460±833 | 9212±1043 | **<0.001** | - |
| BSA (cm^2^) | 4041.83±249.54 | 4282.43±290.35 | **<0.001** | - |
| ﻿**Cardiac morphometry** |  |  |  |  |
| Left atrial area (mm^2^) | 350.20±77.07 | 386.30±78.39 | **0.034** | 0.925 |
| Right atrial area (mm^2^) | 332.66±62.46 | 349.22±62.27 | 0.222 | 0.988 |
| LV area (mm^2^) | 691.97±106.98 | 755.33±133.14 | **0.017** | 0.572 |
| RV area (mm^2^) | 525.37±108.82 | 579.03±95.03 | **0.017** | 0.183 |
| LVSI mid | 2.15 (1.97, 2.27) | 2.13±0.31 | 0.518 | 0.484 |
| LVSI base | 1.77±0.18 | 1.74±0.23 | 0.480 | 0.861 |
| RVSI mid | 2.65±0.36 | 2.56±0.37 | 0.228 | 0.529 |
| RVSI base | 1.80±0.22 | 1.75±0.22 | 0.355 | 0.524 |
| Relative LV wall thickness | 0.24 (0.22, 0.28) | 0.24±0.03 | 0.145 | 0.859 |
| Relative interventricular septal thickness | 0.27 (0.25, 0.29) | 0.26±0.03 | 0.291 | 0.631 |
| Relative RV wall thickness | 0.18±0.05 | 0.19±0.05 | 0.239 | 0.196 |
| **Systolic function** |  |  |  |  |
| LVEF (%) | 65.18±4.79 | 67.90 (62.20, 70.05) | 0.316 | 0.850 |
| RVEF (%) | 67.93±5.47 | 67.27±6.52 | 0.607 | 0.995 |
| Left stroke volume (ml) | 12.10±2.26 | 13.02±2.74 | 0.089 | 0.819 |
| Right stroke volume (ml) | 9.06±2.07 | 10.72±2.79 | **0.002** | 0.145 |
| ﻿Left cardiac output (ml/min) | 1606.61±283.76 | 1681.37±380.76 | 0.302 | 0.812 |
| Right cardiac output (ml/min) | 1177.03±246.38 | 1333.81±352.49 | **0.021** | 0.265 |
| ﻿Mitral ring displacement (mm) | 9.08±1.51 | 9.16 (8.54, 10.09) | 0.292 | 0.657 |
| Tricuspid ring displacement (mm) | 15.07±1.81 | 15.00±2.78 | 0.888 | 0.178 |
| ﻿Mitral S′ (cm/s) | 6.59±1.12 | 6.87±1.46 | 0.319 | 0.362 |
| ﻿Tricuspid S′ (cm/s) | 9.25±1.30 | 9.48±2.23 | 0.563 | 0.516 |
| **Diastolic function** |  |  |  |  |
| Mitral E/A | 1.27 (1.03, 1.48) | 1.27 (1.09, 1.47) | 0.641 | 0.928 |
| Tricuspid E/A | 1.91±0.62 | 1.70±0.66 | 0.119 | 0.108 |
| ﻿Mitral E deceleration time (ms) | 91.91±21.29 | 95.78±22.47 | 0.415 | 0.731 |
| ﻿Tricuspid E deceleration time (ms) | 99.56±20.82 | 106.66±27.11 | 0.174 | 0.708 |
| ﻿Mitral E′ (cm/s) | 10.70±1.87 | 11.14±2.37 | 0.349 | 0.323 |
| Mitral A′ (cm/s) | 8.45±2.65 | 8.40 (6.86, 10.00) | 0.462 | 0.573 |
| Tricuspid E′ (cm/s) | 10.50 (9.30, 12.25) | 10.90 (9.24, 12.60) | 0.348 | 0.811 |
| ﻿Tricuspid A′ (cm/s) | 10.50±3.39 | 8.77 (6.99, 11.60) | 0.968 | 0.822 |
| ﻿Left isovolumic relaxation time (ms) | 53.84±9.53 | 48.00 (45.00, 59.00) | 0.433 | 0.493 |
| **Global cardiac function** |  |  |  |  |
| Left myocardial performance index | 0.20 (0.16, 0.27) | 0.25±0.12 | 0.561 | 0.529 |
| Right myocardial performance index | 0.15 (0.09, 0.25) | 0.17 (0.13, 0.24) | 0.565 | 0.348 |
| **Deformation analysis** |  |  |  |  |
| Frame rate (fps) | 107.2±8.0 | 101.2±9.2 | **0.002** | - |
| Heart rate (bpm) | 132.3±12.7 | 133.0 (120.5, 137.5) | 0.183 | 0.592 |
| Systolic deformation |  |  |  |  |
| LV GLS (%) | -23.52±1.67 | -22.68±1.32 | **0.012** | 0.068 |
| LV GLS rate S (﻿ s^-1^) | -3.79±0.49 | -3.53±0.47 | **0.013** | 0.423 |
| LV global longitudinal T2P strain (ms) | 212.84±13.32 | 211.56±14.31 | 0.668 | 0.695 |
| LV GCS (%) | -26.26±2.41 | -25.66±2.90 | 0.297 | 0.400 |
| LV GCS rate S (﻿ s^-1^) | -4.82±0.82 | -4.47±0.59 | **0.028** | 0.899 |
| LV global circumferential T2P strain (ms) | 199.82±17.46 | 198.17±15.20 | 0.643 | 0.904 |
| RV GLS (%) | -33.04±4.59 | -32.88±3.45 | 0.849 | 0.376 |
| RV GLS rate S (﻿ s^-1^) | -5.81 (-7.26, -5.13) | -5.29 (-6.61, -4.65) | 0.087 | 0.269 |
| RV global longitudinal T2P strain (ms) | 200.22±26.38 | 202.10±28.36 | 0.751 | 0.279 |
| Diastolic deformation |  |  |  |  |
| LV GLS rate E (﻿ s^-1^) | 5.42±0.75 | 4.96±0.72 | **0.005** | 0.431 |
| LV GLS rate A (﻿ s^-1^) | 3.82±0.79 | 3.55±0.76 | 0.110 | 0.575 |
| LV GCS rate E (﻿ s^-1^) | 5.74±1.03 | 5.90±1.17 | 0.515 | 0.075 |
| LV GCS rate A (﻿ s^-1^) | 3.86 (3.11, 4.82) | 3.76±0.94 | 0.217 | 0.531 |
| RV GLS rate E (﻿ s^-1^) | 5.20±2.10 | 5.02 (4.04, 5.74) | 0.816 | 0.157 |
| RV GLS rate A (﻿ s^-1^) | 6.48±1.93 | 5.85 (4.95, 7.12) | 0.654 | 0.531 |
| Dyssynchrony |  |  |  |  |
| Longitudinal strain SD_t18S_ (ms) | 28.84±5.88 | 28.17±7.33 | 0.638 | 0.936 |
| Circumferential strain SD_t18S_ (ms) | 23.42±8.70 | 26.34±8.47 | 0.119 | 0.114 |

ART, pregnancy conceived by assisted reproductive technologies; BSA, body surface area; GCS, global circumferential strain; GLS, global longitudinal strain; LV, left ventricle; LVEF, left ventricular ejection fraction; LVSI, left ventricular sphericity index; RV, right ventricle; RVEF, right ventricular ejection fraction; RVSI, right ventricular sphericity index; SC, spontaneous conception; SD_t18S_, standard deviation of the time to peak systolic strain of 18 segments; T2P, time to peak.

*P values calculated by linear regression adjusted for age at scan, BSA, parity, sex of offspring, birth weight, gestational diabetes, and mean uterine artery pulsatility index. For LV/RV deformation parameters, P values were additionally adjusted for frame rate and LV or RV area.

Table S5. Baseline and perinatal characteristics of controls and fetuses conceived by IVF and ICSI

| Characteristics | SC (n=85) | IVF (n=48) | ICSI (n=38) | P |
| --- | --- | --- | --- | --- |
| **Maternal characteristics** |  |  |  |  |
| Age (y) | 33.0 (31.5, 36.0) | 34.0 (32.0, 36.0) | 34.0 (31.0, 37.0) | 0.948 |
| BMI (kg/m^2^) | 26.11±3.44 | 26.77±4.31 | 25.56 (24.14, 27.82) | 0.850 |
| Han, % (n) | 85.88 (73) | 91.67 (44) | 78.95 (30) | 0.241 |
| Nulliparity, % (n) | 56.47 (48) | 87.50 (42) | 94.74 (36) | **<0.001** |
| Family cardiovascular history, % (n)﻿† | 57.65 (49) | 52.08 (25) | 42.11 (16) | 0.279 |
| Low socioeconomic level, % (n) | 18.82 (16) | 20.83 (10) | 39.47 (15) | **0.039** |
| University education, % (n) | 87.06 (74) | 68.75 (33) | 71.05 (27) | **0.022** |
| **Paternal characteristics** |  |  |  |  |
| Age (y) | 34.8±4.3 | 35.5±3.2 | 36.0±5.1 | 0.304 |
| BMI (kg/m^2^) | 25.77±3.34 | 25.78±3.87 | 24.72±2.93 | 0.247 |
| Han, % (n) | 88.24 (75) | 87.50 (42) | 89.47 (34) | 0.960 |
| Cigarette smoker, % (n) | 34.12 (29) | 52.08 (25) | 34.21 (13) | 0.097 |
| Family cardiovascular history, % (n)﻿† | 57.65 (49) | 52.08 (25) | 52.63 (20) | 0.782 |
| Low socioeconomic level, % (n) | 5.88 (5) | 14.58 (7) | 13.16 (5) | 0.174 |
| University education, % (n) | 85.88 (73) | 70.83 (34) | 68.42 (26) | **0.039** |
| ﻿**Pregnancy complications, % (n)** |  |  |  |  |
| Preeclampsia | 4.71 (4) | 4.17 (2) | 13.16 (5) | 0.205 |
| Gestational diabetes | 23.53 (20) | 10.42 (5) | 28.95 (11) | 0.082 |
| ﻿Placenta previa | 2.35 (2) | 2.08 (1) | 5.26 (2) | 0.710 |
| Obstetric cholestasis | 0 (0) | 0 (0) | 0 (0) | 1.000 |
| Prenatal corticoid exposure | 1.18 (1) | 2.08 (1) | 2.63 (1) | 0.791 |
| **Delivery data** |  |  |  |  |
| Gestational age at delivery (wk) | 39.6±1.0 | 39.5±0.9 | 39.6±1.0 | 0.863 |
| Cesarean section, % (n) | 48.24 (41) | 77.08 (37) | 65.79 (25) | **0.004** |
| Male, % (n) | 47.06 (40) | 58.33 (28) | 42.11 (16) | 0.283 |
| Birth length (cm) | 51.0 (49.5, 52.0) | 51.0 (50.0, 52.0) | 51.0 (50.0, 52.0) | 0.836 |
| Birth weight (g) | 3380±280 | 3448±314 | 3518 (3115, 3699) | 0.359 |

BMI, body mass index; ICSI, intracytoplasmic sperm injection; IVF, in vitro fertilization; SC, spontaneous conception.

†Family cardiovascular history is defined as the existence of congenital heart disease, coronary disease, hypertension, diabetes, hypercholesterolemia or stroke in men<55 years and women<65 years.

Table S6. Baseline and perinatal characteristics of controls and fetuses conceived by fresh ET and FET

| Characteristics | SC (n=85) | fresh ET (n=21) | FET (n=67) | P |
| --- | --- | --- | --- | --- |
| **Maternal characteristics** |  |  |  |  |
| Age (y) | 33.0 (31.5, 36.0) | 33.3±2.4 | 34.0 (31.0, 36.0) | 0.592 |
| BMI (kg/m^2^) | 26.11±3.44 | 27.26±4.26 | 25.59 (24.02, 28.04) | 0.410 |
| Han, % (n) | 85.88 (73) | 91.67 (19) | 78.95 (53) | 0.241 |
| Nulliparity, % (n) | 56.47 (48) | 100.00 (21) | 86.57 (58) | **<0.001** |
| Family cardiovascular history, % (n)† | 57.65 (49) | 47.62 (10) | 49.25 (33) | 0.507 |
| Low socioeconomic level, % (n) | 18.82 (16) | 33.33 (7) | 26.87 (18) | 0.277 |
| University education, % (n) | 87.06 (74) | 61.90 (13) | 73.13 (49) | **0.016** |
| **Paternal characteristics** |  |  |  |  |
| Age (y) | 34.8±4.3 | 35.1±3.0 | 35.9±4.4 | 0.278 |
| BMI (kg/m^2^) | 25.77±3.34 | 25.20±3.68 | 25.36±3.44 | 0.676 |
| Han, % (n) | 88.24 (75) | 87.50 (18) | 89.47 (60) | 0.960 |
| Cigarette smoker, % (n) | 34.12 (29) | 28.57 (6) | 47.76 (32) | 0.137 |
| Family cardiovascular history, % (n)† | 57.65 (49) | 47.62 (10) | 55.22 (37) | 0.709 |
| Low socioeconomic level, % (n) | 5.88 (5) | 9.52 (2) | 14.93 (10) | 0.177 |
| University education, % (n) | 85.88 (73) | 57.14 (12) | 74.63 (50) | **0.012** |
| ﻿**Pregnancy complications, % (n)** |  |  |  |  |
| Preeclampsia | 4.71 (4) | 9.52 (2) | 8.96 (6) | 0.445 |
| Gestational diabetes | 23.53 (20) | 19.05 (4) | 17.91 (12) | 0.683 |
| ﻿Placenta previa | 2.35 (2) | 9.52 (2) | 2.99 (2) | 0.262 |
| Obstetric cholestasis | 0 (0) | 0 (0) | 0 (0) | 1.000 |
| Prenatal corticoid exposure | 1.18 (1) | 4.76 (1) | 1.49 (1) | 0.496 |
| **Delivery data** |  |  |  |  |
| Gestational age at delivery (wk) | 39.6±1.0 | 39.5±1.0 | 39.5±0.9 | 0.922 |
| Cesarean section, % (n) | 48.24 (41) | 57.14 (12) | 77.61 (52) | **0.001** |
| Male, % (n) | 47.06 (40) | 47.62 (10) | 53.73 (36) | 0.701 |
| Birth length (cm) | 51.0 (49.5, 52.0) | 51.0 (50.0, 52.0) | 51.0 (50.0, 52.0) | 0.843 |
| Birth weight (g) | 3380±280 | 3405±375 | 3461±297 | 0.228 |

BMI, body mass index; ET, embryo transfer; FET, frozen embryo transfer; SC, spontaneous conception.

†Family cardiovascular history is defined as the existence of congenital heart disease, coronary disease, hypertension, diabetes, hypercholesterolemia or stroke in men<55 years and women<65 years.

Table S7. Fetal assessment of controls and fetuses conceived by IVF and ICSI

| Characteristics | SC (n=85) | IVF (n=48) | ICSI (n=38) | P |
| --- | --- | --- | --- | --- |
| Gestational age at scan (wk) | 29.7 (28.7, 31.3) | 30.1±1.4 | 29.5 (28.9, 30.2) | 0.526 |
| Estimated fetal weight (g) | 1460 (1265, 1733) | 1522±272 | 1348 (1275, 1650) | 0.492 |
| Placental data |  |  |  |  |
| Middle cerebral artery PI | 1.97 (1.84, 2.17) | 1.92±0.32 | 1.87±0.33 | 0.154 |
| Umbilical artery PI | 1.04±0.17 | 1.00±0.16 | 1.06±0.17 | 0.298 |
| Cerebroplacental ratio | 1.89 (1.71, 2.12) | 1.86 (1.66, 2.10) | 1.79±0.34 | 0.090 |
| Aortic isthmus PI | 2.68±0.35 | 2.65±0.37 | 2.70±0.33 | 0.735 |
| Ductus venosus PI | 0.54 (0.45, 0.69) | 0.62 (0.49, 0.81) | 0.54 (0.42, 0.65) | 0.071 |
| Mean uterine artery PI | 0.85±0.21 | 0.75±0.20* | 0.74±0.21* | **0.005** |
| **Cardiac morphometry** |  |  |  |  |
| Left atrial area (mm^2^) | 167.99±35.70 | 163.24±32.78 | 168.84±26.56 | 0.667 |
| Right atrial area (mm^2^) | 173.73±31.48 | 177.10±35.29 | 171.11±28.70 | 0.684 |
| LV area (mm^2^) | 244.25±47.13 | 262.03±45.47 | 259.55±46.24 | 0.065 |
| RV area (mm^2^) | 221.14±42.60 | 239.60±36.98* | 230.05±40.58 | **0.043** |
| LVSI mid | 2.45±0.39 | 2.27±0.28* | 2.30±0.39 | **0.009** |
| LVSI base | 2.50±0.40 | 2.34±0.27* | 2.48±0.40 | **0.047** |
| RVSI mid | 2.12 (1.97, 2.41) | 2.11±0.33 | 2.18±0.29 | 0.249 |
| RVSI base | 2.11±0.28 | 2.11±0.28 | 2.20±0.20 | 0.164 |
| Relative LV wall thickness | 0.36±0.05 | 0.34±0.04 | 0.35±0.05 | 0.106 |
| Relative interventricular septal thickness | 0.37±0.06 | 0.36±0.05 | 0.36±0.06 | 0.329 |
| Relative RV wall thickness | 0.34±0.05 | 0.33±0.06 | 0.34±0.04 | 0.533 |
| ﻿Cardiothoracic ratio | 0.29 (0.27, 0.32) | 0.28 (0.26, 0.32) | 0.29±0.03 | 0.149 |
| **Systolic function** |  |  |  |  |
| LVEF (%) | 63.43±5.22 | 63.64±4.16 | 63.38±4.86 | 0.962 |
| RVEF (%) | 58.61±6.22 | 58.75 (56.43, 62.08) | 59.53±5.50 | 0.451 |
| Left stroke volume (ml) | 2.46±0.71 | 2.55±0.85 | 2.22 (1.66, 2.89) | 0.389 |
| Right stroke volume (ml) | 3.58 (3.01, 3.94) | 3.82±1.08 | 3.52±0.78 | 0.483 |
| Left cardiac output (ml/min) | 352.25±97.65 | 353.07±119.51 | 322.94 (248.62, 394.88) | 0.632 |
| Right cardiac output (ml/min) | 528.57 (446.15, 574.26) | 559.48±148.02 | 506.65±105.71 | 0.340 |
| Mitral ring displacement (mm) | 6.75±1.81 | 6.44±1.93 | 5.77±1.77* | **0.026** |
| ﻿Tricuspid ring displacement (mm) | 8.30±1.81 | 7.82±1.90 | 7.37±1.61* | **0.027** |
| ﻿Mitral S′ (cm/s) | 5.74±0.82 | 5.68±1.11 | 5.90 (5.27, 6.31) | 0.538 |
| Tricuspid S′ (cm/s) | 6.29 (5.71, 6.96) | 6.05 (5.71, 6.75) | 6.14 (5.66, 6.80) | 0.819 |
| **Diastolic function** |  |  |  |  |
| Mitral E/A | 0.72±0.08 | 0.75±0.10 | 0.73±0.11 | 0.096 |
| Tricuspid E/A | 0.73 (0.68, 0.79) | 0.72 (0.67, 0.80) | 0.74±0.09 | 0.452 |
| Mitral E deceleration time (ms) | 40.00 (32.00, 48.00) | 46.96±17.13 | 41.37±14.28 | 0.262 |
| ﻿Tricuspid E deceleration time (ms) | 28.00 (21.00, 36.00) | 31.00 (22.50, 42.00) | 29.63±10.37 | 0.313 |
| ﻿Mitral E′ (cm/s) | 5.59±0.79 | 5.70±0.93 | 6.00±0.75* | **0.042** |
| Mitral A′ (cm/s) | 8.35±1.69 | 8.20±1.70 | 8.22 (7.33, 9.02) | 0.777 |
| Tricuspid E′ (cm/s) | 6.77 (5.61, 8.51) | 6.63 (5.90, 8.39) | 7.28±1.93 | 0.940 |
| Tricuspid A′ (cm/s) | 11.04±1.81 | 10.86±2.44 | 10.83±2.46 | 0.702 |
| Left isovolumic relaxation time (ms) | 48.16±9.44 | 46.63±9.41 | 44.79±7.59 | 0.156 |
| **Global cardiac function** |  |  |  |  |
| Left myocardial performance index | 0.45±0.14 | 0.41 (0.32, 0.53) | 0.38 (0.30, 0.54) | 0.937 |
| Right myocardial performance index | 0.38±0.12 | 0.38±0.15 | 0.37±0.17 | 0.921 |
| **Deformation analysis** |  |  |  |  |
| Frame rate (fps) | 97.0 (92.0, 103.5) | 95.0 (89.3, 102.8) | 93.0 (89.0, 101.5) | 0.194 |
| Heart rate (bpm) | 145.1±7.1 | 144.1±5.7 | 144.5±7.6 | 0.675 |
| Systolic deformation |  |  |  |  |
| LV GLS (%) | -20.65±1.88 | -19.60±1.86* | -19.39±1.92* | **<0.001** |
| LV GLS rate S (﻿ s^-1^) | -3.58±0.39 | -3.32±0.39* | -3.32±0.33* | **<0.001** |
| LV global longitudinal T2P strain (ms) | 209.60±13.92 | 215.29±14.14 | 214.05±15.04 | 0.059 |
| LV GCS (%) | -19.83±2.49 | -18.64±2.66* | -19.36±2.88 | **0.045** |
| LV GCS rate S (﻿ s^-1^) | -3.89±0.56 | -3.69±0.54 | -3.69±0.54 | 0.059 |
| LV global circumferential T2P strain (ms) | 208.72±12.95 | 207.85±10.86 | 206.18±14.82 | 0.601 |
| RV GLS (%) | -21.58±3.64 | -20.21±4.26 | -20.91±3.28 | 0.130 |
| RV GLS rate S (﻿ s^-1^) | -3.41±0.76 | -3.26±0.77 | -3.22±0.87 | 0.397 |
| RV global longitudinal T2P strain (ms) | 196.00 (185.00, 217.50) | 197.88±26.26 | 207.66±24.17 | 0.201 |
| Diastolic deformation |  |  |  |  |
| LV GLS rate E (﻿ s^-1^) | 3.22±0.47 | 3.17±0.49 | 3.14±0.39 | 0.645 |
| LV GLS rate A (﻿ s^-1^) | 3.85±0.57 | 3.62±0.48 | 3.64±0.58 | **0.034** |
| LV GCS rate E (﻿ s^-1^) | 3.53±0.51 | 3.34±0.57 | 3.49 (2.93, 3.72) | 0.244 |
| LV GCS rate A (﻿ s^-1^) | 3.46±0.67 | 3.26±0.62 | 3.07±0.56* | **0.007** |
| RV GLS rate E (﻿ s^-1^) | 3.19±1.19 | 2.70 (2.07, 3.36) | 3.09±0.95 | 0.365 |
| RV GLS rate A (﻿ s^-1^) | 3.60 (2.93, 4.30) | 3.72±1.42 | 3.80±1.21 | 0.939 |
| Dyssynchrony |  |  |  |  |
| Longitudinal strain SD_t18S_ (ms) | 26.41±6.59 | 27.84±6.81 | 26.98±5.93 | 0.478 |
| Circumferential strain SD_t18S_ (ms) | 22.75 (17.62, 27.74) | 24.80±7.30 | 23.03±6.59 | 0.530 |

GCS, global circumferential strain; GLS, global longitudinal strain; ICSI, intracytoplasmic sperm injection; IVF, in vitro fertilization; LV, left ventricle; LVEF, left ventricular ejection fraction; LVSI, left ventricular sphericity index; PI, pulsatility index; RV, right ventricle; RVEF, right ventricular ejection fraction; RVSI, right ventricular sphericity index; SC, spontaneous conception; SD_t18S_, standard deviation of the time to peak systolic strain of 18 segments; T2P, time to peak.

*p<0.05 compared with the SC group by Bonferroni correction.

Table S8. Fetal assessment of controls and fetuses conceived by fresh ET and FET

| Characteristics | SC (n=85) | fresh ET (n=21) | FET (n=67) | P |
| --- | --- | --- | --- | --- |
| Gestational age at scan (wk) | 29.7 (28.7, 31.3) | 29.9±1.2 | 30.0±1.3 | 0.974 |
| Estimated fetal weight (g) | 1460 (1265, 1733) | 1483±263 | 1472 (1298, 1673) | 0.872 |
| Placental data |  |  |  |  |
| Middle cerebral artery PI | 1.97 (1.84, 2.17) | 1.86±0.32 | 1.91±0.32 | 0.170 |
| Umbilical artery PI | 1.04±0.17 | 1.05±0.19 | 1.02±0.15 | 0.647 |
| Cerebroplacental ratio | 1.89 (1.71, 2.12) | 1.80±0.32 | 1.86 (1.66, 2.07) | 0.300 |
| Aortic isthmus PI | 2.68±0.35 | 2.72±0.35 | 2.67±0.36 | 0.884 |
| Ductus venosus PI | 0.54 (0.45, 0.69) | 0.52 (0.47, 0.70) | 0.56 (0.47, 0.75) | 0.508 |
| Mean uterine artery PI | 0.85±0.21 | 0.82±0.21 | 0.72±0.19* | **0.001** |
| **Cardiac morphometry** |  |  |  |  |
| Left atrial area (mm^2^) | 167.99±35.70 | 166.72±24.11 | 166.42±32.11 | 0.956 |
| Right atrial area (mm^2^) | 173.73±31.48 | 175.03±29.90 | 175.18±33.52 | 0.959 |
| LV area (mm^2^) | 244.25±47.13 | 249.70±45.06 | 266.29±45.85* | **0.015** |
| RV area (mm^2^) | 221.14±42.60 | 229.70±31.77 | 238.11±40.54* | **0.040** |
| LVSI mid | 2.45±0.39 | 2.36±0.35 | 2.27±0.33* | **0.007** |
| LVSI base | 2.50±0.40 | 2.49±0.39 | 2.38±0.32 | 0.117 |
| RVSI mid | 2.12 (1.97, 2.41) | 2.10±0.32 | 2.16±0.33 | 0.371 |
| RVSI base | 2.11±0.28 | 2.11±0.25 | 2.17±0.26 | 0.305 |
| Relative LV wall thickness | 0.36±0.05 | 0.36±0.05 | 0.34±0.04* | **0.028** |
| Relative interventricular septal thickness | 0.37±0.06 | 0.38±0.05 | 0.35±0.05 | 0.091 |
| Relative RV wall thickness | 0.34±0.05 | 0.34±0.06 | 0.34±0.05 | 0.895 |
| ﻿Cardiothoracic ratio | 0.29 (0.27, 0.32) | 0.28 (0.27, 0.30) | 0.29±0.03 | 0.133 |
| **Systolic function** |  |  |  |  |
| LVEF (%) | 63.43±5.22 | 63.76±4.80 | 63.37±4.34 | 0.950 |
| RVEF (%) | 58.61±6.22 | 60.10 (55.15, 61.45) | 58.86±6.38 | 0.813 |
| Left stroke volume (ml) | 2.46±0.71 | 2.70±0.91 | 2.23 (1.71, 2.99) | 0.213 |
| Right stroke volume (ml) | 3.58 (3.01, 3.94) | 3.64±0.94 | 3.71±0.96 | 0.893 |
| Left cardiac output (ml/min) | 352.25±97.65 | 365.12 (271.24, 408.31) | 325.30 (247.32, 421.79) | 0.251 |
| Right cardiac output (ml/min) | 528.57 (446.15, 574.26) | 539.97±130.66 | 536.36±133.00 | 0.940 |
| Mitral ring displacement (mm) | 6.75±1.81 | 6.77±1.81 | 5.92±1.85* | **0.015** |
| ﻿Tricuspid ring displacement (mm) | 8.30±1.81 | 8.15±1.74 | 7.45±1.75* | **0.013** |
| ﻿Mitral S′ (cm/s) | 5.74±0.82 | 5.72±0.82 | 5.90 (5.13, 6.38) | 0.799 |
| Tricuspid S′ (cm/s) | 6.29 (5.71, 6.96) | 5.90 (5.47, 6.87) | 6.29 (5.80, 6.77) | 0.614 |
| **Diastolic function** |  |  |  |  |
| Mitral E/A | 0.72±0.08 | 0.75±0.08 | 0.74±0.11 | 0.141 |
| Tricuspid E/A | 0.73 (0.68, 0.79) | 0.76 (0.69, 0.83) | 0.73 (0.66, 0.80) | 0.600 |
| Mitral E deceleration time (ms) | 40.00 (32.00, 48.00) | 44.00 (30.00, 50.00) | 44.00 (32.00, 56.00) | 0.575 |
| ﻿Tricuspid E deceleration time (ms) | 28.00 (21.00, 36.00) | 30.00 (24.00, 38.00) | 28.00 (20.00, 40.00) | 0.962 |
| ﻿Mitral E′ (cm/s) | 5.59±0.79 | 5.92±0.69 | 5.82±0.91 | 0.123 |
| Mitral A′ (cm/s) | 8.35±1.69 | 8.58±2.07 | 8.07±1.47 | 0.377 |
| Tricuspid E′ (cm/s) | 6.77 (5.61, 8.51) | 7.28±2.31 | 6.77 (5.90, 8.41) | 0.966 |
| Tricuspid A′ (cm/s) | 11.04±1.81 | 10.91±2.68 | 10.88±2.36 | 0.828 |
| Left isovolumic relaxation time (ms) | 48.16±9.44 | 46.10±9.00 | 45.82±8.50 | 0.254 |
| **Global cardiac function** |  |  |  |  |
| Left myocardial performance index | 0.45±0.14 | 0.40 (0.33, 0.49) | 0.39 (0.30, 0.54) | 0.265 |
| Right myocardial performance index | 0.38±0.12 | 0.38±0.14 | 0.38±0.17 | 1.000 |
| **Deformation analysis** |  |  |  |  |
| Frame rate (fps) | 97.0 (92.0, 103.5) | 96.9±8.4 | 94.0 (89.0, 103.0) | 0.271 |
| Heart rate (bpm) | 145.1±7.1 | 145.5±5.6 | 143.8±6.8 | 0.410 |
| Systolic deformation |  |  |  |  |
| LV GLS (%) | -20.65±1.88 | -19.53±1.93* | -19.56±1.90* | **0.001** |
| LV GLS rate S (﻿ s^-1^) | -3.58±0.39 | -3.28±0.29* | -3.34±0.38* | **<0.001** |
| LV global longitudinal T2P strain (ms) | 209.60±13.92 | 214.10±12.91 | 214.63±14.93 | 0.079 |
| LV GCS (%) | -19.83±2.49 | -18.35±1.86 | -19.15±2.94 | **0.046** |
| LV GCS rate S (﻿ s^-1^) | -3.89±0.56 | -3.56±0.44* | -3.75±0.56 | **0.034** |
| LV global circumferential T2P strain (ms) | 208.72±12.95 | 207.33±12.25 | 206.78±13.18 | 0.648 |
| RV GLS (%) | -21.58±3.64 | -20.68±4.54 | -20.55±3.62 | 0.221 |
| RV GLS rate S (﻿ s^-1^) | -3.41±0.76 | -3.06±0.73 | -3.31±0.84 | 0.200 |
| RV global longitudinal T2P strain (ms) | 196.00 (185.00, 217.50) | 203.90±24.19 | 201.84±26.02 | 0.947 |
| Diastolic deformation |  |  |  |  |
| LV GLS rate E (﻿ s^-1^) | 3.22±0.47 | 3.13±0.37 | 3.17±0.46 | 0.653 |
| LV GLS rate A (﻿ s^-1^) | 3.85±0.57 | 3.64±0.42 | 3.63±0.55* | **0.036** |
| LV GCS rate E (﻿ s^-1^) | 3.53±0.51 | 3.27±0.46 | 3.37 (2.93, 3.75) | 0.235 |
| LV GCS rate A (﻿ s^-1^) | 3.46±0.67 | 3.16±0.59 | 3.17±0.60* | **0.015** |
| RV GLS rate E (﻿ s^-1^) | 3.19±1.19 | 2.80±1.00 | 2.81 (2.09, 3.37) | 0.354 |
| RV GLS rate A (﻿ s^-1^) | 3.60 (2.93, 4.30) | 3.77±1.61 | 3.78±1.23 | 0.992 |
| Dyssynchrony |  |  |  |  |
| Longitudinal strain SD_t18S_ (ms) | 26.41±6.59 | 25.36±4.43 | 27.89±6.88 | 0.206 |
| Circumferential strain SD_t18S_ (ms) | 22.75 (17.62, 27.74) | 26.98±7.61 | 23.23±6.56 | 0.134 |

ET, embryo transfer; FET, frozen embryo transfer; GCS, global circumferential strain; GLS, global longitudinal strain; LV, left ventricle; LVEF, left ventricular ejection fraction; LVSI, left ventricular sphericity index; PI, pulsatility index; RV, right ventricle; RVEF, right ventricular ejection fraction; RVSI, right ventricular sphericity index; SC, spontaneous conception; SD_t18S_, standard deviation of the time to peak systolic strain of 18 segments; T2P, time to peak.

*p<0.05 compared with the SC group by Bonferroni correction.

Table S9. Intra-observer and inter-observer ICCs for LV deformation parameters

| Parameters | Intra-observer | |  | Inter-observer | |
| --- | --- | --- | --- | --- | --- |
|  | Prenatal | Postnatal |  | Prenatal | Postnatal |
| GLS | 0.71 | 0.82 |  | 0.75 | 0.86 |
| Global longitudinal T2P strain | 0.95 | 0.94 |  | 0.94 | 0.95 |
| GLS rate S | 0.59 | 0.88 |  | 0.76 | 0.81 |
| GLS rate E | 0.92 | 0.85 |  | 0.95 | 0.91 |
| GLS rate A | 0.89 | 0.84 |  | 0.86 | 0.89 |
| GCS | 0.82 | 0.95 |  | 0.71 | 0.65 |
| Global circumferential T2P strain | 0.90 | 0.95 |  | 0.90 | 0.90 |
| GCS rate S | 0.95 | 0.95 |  | 0.91 | 0.90 |
| GCS rate E | 0.93 | 0.89 |  | 0.91 | 0.84 |
| GCS rate A | 0.84 | 0.95 |  | 0.68 | 0.94 |

GCS, global circumferential strain; GLS, global longitudinal strain; ICCs, intraclass correlation coefficients; LV, left ventricle; T2P, time to peak.
